# *Pseudomonas aeruginosa* ExoS Induces Intrinsic Apoptosis in Target Host Cells in a Manner That is Dependent on its GAP Domain Activity

**DOI:** 10.1038/s41598-018-32491-2

**Published:** 2018-09-19

**Authors:** Amber Kaminski, Kajal H. Gupta, Josef W. Goldufsky, Ha Won Lee, Vineet Gupta, Sasha H. Shafikhani

**Affiliations:** 1 Department of Medicine, Rush University Medical Center, Chicago, IL USA; 2 Department of Microbial Pathogens and Immunity, Rush University Medical Center, Chicago, IL USA; 3 Cancer Center, Rush University Medical Center, Chicago, IL USA

## Abstract

*Pseudomonas aeruginosa* is a Gram-negative opportunistic pathogen that causes serious infections in immunocompromised individuals and cystic fibrosis patients. ExoS and ExoT are two homologous bifunctional Type III Secretion System (T3SS) virulence factors that induce apoptosis in target host cells. They possess a GTPase Activating Protein (GAP) domain at their N-termini, which share ~76% homology, and an ADP-ribosyltransferase (ADPRT) domain at their C-termini, which target non-overlapping substrates. Both the GAP and the ADPRT domains contribute to ExoT’s cytotoxicity in target epithelial cells, whereas, ExoS-induced apoptosis is reported to be primarily due to its ADPRT domain. In this report, we demonstrate that ExoS/GAP domain is both necessary and sufficient to induce mitochondrial apoptosis. Our data demonstrate that intoxication with ExoS/GAP domain leads to enrichment of Bax and Bim into the mitochondrial outer-membrane, disruption of mitochondrial membrane and release of and cytochrome *c* into the cytosol, which activates initiator caspase-9 and effector caspase-3, that executes cellular death. We posit that the contribution of the GAP domain in ExoS-induced apoptosis was overlooked in prior studies due to its slower kinetics of cytotoxicity as compared to ADPRT. Our data clarify the field and reveal a novel virulence function for ExoS/GAP as an inducer of apoptosis.

## Introduction

*Pseudomonas aeruginosa* is a Gram-negative, opportunistic pathogen which is one of the leading causes of nosocomial pneumonia and respiratory failure, and bacteremia and sepsis in immunocompromised patients^1^. Chronic infection by *P. aeruginosa* is a characteristic of individuals afflicted with cystic fibrosis or individuals with chronic wounds^2–4^. Bacterial infections due to *P. aeruginosa* have also been reported as a complication of AIDS^5–7^.

Amongst the plethora of virulence structures and factors that mediate the pathogenesis of *P. aeruginosa* is a Type III Secretion System (T3SS) apparatus that functions as a conduit, allowing direct translocation of T3SS effector virulence factors into the target host cytoplasm, which paralyze host cellular processes in order to facilitate *P. aeruginosa* infection^8,9^. These T3SS effector proteins mediate tissue damage, inhibit wound healing, and are crucial for the establishment of *P. aeruginosa* infection^2,8,10–12^. To date, four T3SS effector proteins with well-characterized virulence functions have been described in *P. aeruginosa*^8^. ExoU is a potent cytotoxin–present in cytotoxic *P. aeruginosa* strains–with phospholipase A_2_ activity capable of inducing rapid necrotic cytotoxicity in various eukaryotic cells^13,14^. ExoY is a nucleotidyl cyclase edema factor that disrupts barrier integrity and causes tissue edema but has no role in cytotoxicity^15,16^. ExoT alters the actin cytoskeleton, inhibits cytokinesis, induces various forms of apoptotic cell death, and blocks apoptotic compensatory proliferation signaling in target host cells^11,17–22^. Lastly, ExoS is present in invasive *P. aeruginosa* strains, which works to rearrange the actin cytoskeleton and induces apoptosis in target host cells^23–26^.

ExoS and ExoT are homologous bifunctional proteins that possess a GTPase-activating protein (GAP) domain at their N-termini, which inactivate small GTPases, namely RhoA, Rac1, and Cdc42^24,25^, and an ADP-ribosyltransferase domain (ADPRT) at their C-termini, which target non-overlapping cellular substrates^8,10^. Both GAP and the ADPRT domains contribute to ExoT-induced cytotoxicity^19–21,27^. Through ADP-ribosylating Crk adaptor protein, the ADPRT domain of ExoT disrupts integrin survival signaling, thus causing a form of apoptosis known as anoikis apoptosis^20^. The GAP domain of ExoT causes typical caspase-9 dependent intrinsic (mitochondrial) apoptosis by disrupting the mitochondrial outer-membrane^21^. In contrast to ExoT, ExoS-induced apoptosis has been primarily attributed to its ADPRT domain activity^23,28–31^. ExoS-intoxicated cells exhibit signs of both caspase-9 dependent intrinsic apoptosis and death receptor-mediated caspase-8 dependent extrinsic apoptosis. ExoS intoxication has been demonstrated to result in cytochrome *c* release into the cytosol and initiator caspase-9 and effector caspase-3 activation (all hallmarks of typical intrinsic apoptosis^32,33^), leading to the demise of the ExoS-intoxicated target host cell^28,29^. ExoS/ADPRT intoxication has also been shown to result in the activation of initiator caspase-8 in a manner that is dependent on the Fas-associated protein with death domain (FADD) adaptor protein, although ExoS-induced apoptosis was shown to be independent of Fas death receptor and caspase-8 activities^30^. ExoS has also been shown to induce DNA double strand breaks in macrophages in a manner that is dependent on its ADPRT domain activity^34^. Since ExoS/GAP and ExoT/GAP domains share over 70% sequence homology and their activities appear to be biochemically and biologically identical^8,17,26^, we sought to re-examine the contribution of the GAP domain in ExoS-induced apoptosis.

Our data in this report demonstrate that ExoS/GAP is necessary and sufficient to cause intrinsic/mitochondrial apoptosis. ExoS/GAP intoxication (not ExoS/ADPRT domain) leads to mobilization and enrichment of Bax and Bim into the mitochondrial outer-membrane, resulting in disruption of mitochondrial outer-membrane and release of cytochrome *c* into the cytosol, which in turn leads to initiator caspase-9 activation, that subsequently activates effector caspase-3 which executes apoptotic cell death. Our data further show that while the low-level of ExoS-induced cytotoxicity (15–20%) that occurs early (within the first 5 h post-infection) is primarily due to the ADPRT domain activity, the GAP domain is a major contributor to ExoS-induced apoptosis at later timepoints (15–20 h) when ExoS-induced cytotoxicity reaches its maximum level. These studies clarify the field and reveal a previously unappreciated role for the GAP domain of ExoS as an inducer of apoptosis.

## Results

### ExoS-induced mitochondrial disruption in the target host cell is due to its GAP domain activity

Previously, it has been demonstrated that ExoS intoxication results in mitochondrial outer-membrane disruption and cytochrome *c* release into the cytosol in a manner that is dependent on its ADPRT domain activity^29^. Recent studies however, demonstrated that intoxication with the GAP domain of ExoT results in mitochondrial damage and cytochrome *c* release into the cytosol of target epithelial cells^21^. Prompted by the high degree of homology (>70%) between ExoS/GAP and ExoT/GAP domains^24,25^, we re-examined the contributions of the GAP and the ADPRT domains to mitochondrial membrane disruption and cytochrome *c* release caused by ExoS. To this end, we treated HeLa cells with PBS (Mock) or infected them with *exoU-* and *exoT*-deleted (∆U∆T) PA103 strains, expressing wildtype ExoS (∆U∆T/ExoS), ExoS with functional GAP and mutant ADPRT (∆U∆T/ExoS (G^+^A^−^)), ExoS with functional ADPRT and mutant GAP (∆U∆T/ExoS (G^−^A^+^)); ExoS with mutant GAP and mutant ADPRT (∆U∆T/ExoS (G^−^A^−^)), or T3SS mutant *pscJ::gent*^*R*^ (*pscJ*) (See Table S1 and Methods for information regarding constructed strains). We chose PA103 strain because in this strain genetic background, the T3SS-induced apoptosis predominates over the T3SS-independent cytotoxins, such as elastase, rhamnolipid, pyocyanin, azurin, Las A protease, and 3-oxo-C12-HSL, some of which have also been shown to induce apoptosis^12,19–21,27,35–39^.

**Figure 1:**
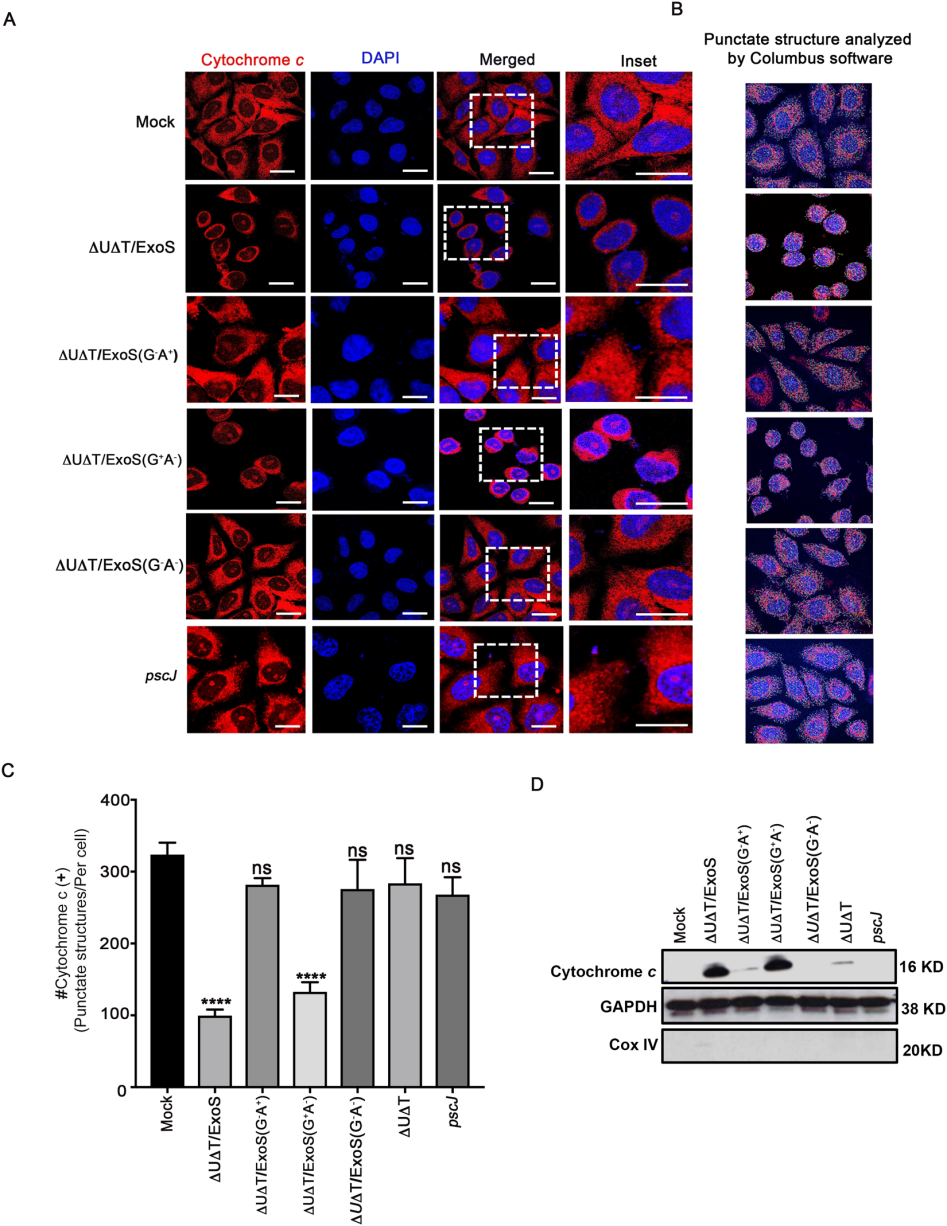
ExoS-induced mitochondrial membrane disruption and cytochrome *c* release into the cytosol is dependent on its GAP domain activity. HeLa cells were infected with ExoS-expressing ∆U∆T/ExoS, ExoS/GAP-expressing ∆U∆T/ExoS (G^+^A^−^), ExoS/ADPRT-expressing ∆U∆T/ExoS (G^−^A^+^) strain, or the T3SS mutant (*pscJ*) at a MOI of 10. (**A**) Five hours following infection, cells were fixed and stained with DAPI nuclear dye (blue) and cytochrome *c* (red) to inspect the impact of the ExoS domains on mitochondria. Representative images are shown. Scale bar represents 10 μm. (**B**,**C**) Columbus software was used to determine the number of intact mitochondria (defined as cytochrome *c* positive punctate structures and represented by different colored circles in these images) per cell. Representative images are shown in (**B**) and the corresponding data are shown in (**C**). Results were tabulated from 5 random fields, compared to Mock, and presented as the Mean ± SD (****p < 0.0001, One-way ANOVA). (**D**) The cytoplasmic fractions of the aforementioned infected HeLa cells were assessed for their cytochrome *c* contents by Western blotting, following a 5 h infection as described above. GAPDH and Cox IV were used as loading controls for cytoplasmic and mitochondrial fractions respectively (Equal amounts of proteins were loaded on 3 gels and run simultaneously. The gels were then probed with either cytochrome *c*, GAPDH, or Cox IV. Each experiment was repeated at least 3 times and a representative blot of each is shown).

Following a 5 h infection, infected HeLa cells were fixed and stained for cytochrome *c* to assess mitochondrial health by immunofluorescent (IF) microscopy. HeLa cells infected with ExoS or ExoS (G^+^A^−^)-expressing *P. aeruginosa* strains exhibited diffuse cytochrome *c* staining (Fig. 1A), which is indicative of mitochondrial disruption^21,40^. In contrast, uninfected HeLa cells (Mock) or HeLa cells infected with ∆U∆T/ExoS (G^−^A^+^), ∆U∆T/ExoS (G^−^A^−^), or the T3SS mutant *pscJ*, exhibited punctate globular staining of cytochrome *c*, which is indicative of intact healthy mitochondria^21,40^. To quantify the impact of ExoS/GAP and ExoS/ADPRT domain activities on mitochondrial health, we analyzed the number of intact (healthy) mitochondria (defined based on their globular structures), by Columbus software (Methods). Consistent with IF microscopy results, HeLa cells infected with ΔUΔT/ExoS or ΔUΔT/ExoS (G^+^A^−^) showed significant reductions in the number of intact mitochondria (Fig. 1B,C).

To rule out the possibility that cell rounding which occurred in response to GAP domain activity, due to its inhibitory effect on RhoA small GTPase^26^, did not skew the mitochondrial analyses by Columbus software, we assessed the level of cytochrome *c* in the cytosolic fraction of infected cells by Western blotting, following a 5 h infection with the aforementioned bacterial strains. Consistent with IF microscopy data in Fig. 1A–C, HeLa cells infected with ExoS or ExoS (G^+^A^−^)- expressing *P. aeruginosa* strains contained substantially higher levels of cytochrome *c* in their cytosolic fractions than those infected with strains lacking GAP domain activity (Fig. 1D). These data indicated that disruption of mitochondrial membrane and release of cytochrome *c* into target host cell cytosol by ExoS is primarily due to its GAP domain activity.

To assess if ExoS/GAP domain was sufficient to cause mitochondrial membrane damage and cytochrome *c* release into the cytosol, we constructed pIRES2-EGFP mammalian expression vectors containing full length ExoS with functional GAP and mutant ADPRT (pExoS (G^+^A^−^)), full length ExoS with mutant GAP and mutant ADPRT (pExoS (G^−^A^−^)), and truncated ExoS containing either functional or mutant GAP domain (pExoS (G^+^) and pExoS (G^−^) respectively), all fused directly at their C-termini to GFP (Fig. S1A, Table S1 and Methods). HeLa cells were then transfected with these mammalian expression vectors, as we previously described^20,21^. Twenty hours after transfection, HeLa cells were fixed and then stained for GFP (to identify transfected cells) and cytochrome *c* (to assess mitochondrial health). The transfection efficiencies of the indicated vectors were similar, although some degradation products of transfected GFP fusion proteins were observed (Fig. S1B). Importantly, cells transfected with vectors containing functional GAP (pExoS (G^+^A^−^) or pExoS (G^+^)) displayed diffuse cytochrome *c* staining (Fig. S1C), indicative of disrupted mitochondria in cells undergoing intrinsic apoptosis^21,41^. As expected, there were also significant reductions in the number of cytochrome *c* positive globular intact mitochondria, as determined by Columbus software (Fig. S1D,E). In contrast, cells that were transfected with GFP, ExoS (G^−^A^−^), or ExoS (G^−^) exhibited punctate cytochrome *c* staining, which is consistent with the staining pattern seen in intact healthy mitochondria^21,41^. To further corroborate these data, we repeated the transfection studies and evaluated the impact of ExoS/GAP on mitochondrial health, using the mitochondrial marker MitoTracker^42^. In line with the cytochrome *c* data (Fig. S1), transfection with vectors containing functional GAP (pExoS (G^+^A^−^) or pExoS (G^+^)) resulted in diffuse MitoTracker staining and a significant reduction in the number of MitoTracker positive globular intact mitochondria, as determined by Columbus software (Fig. S2). Collectively, these data indicated that ExoS-induced mitochondrial disruption and cytochrome *c* release is primarily dependent on its GAP domain activity, not the ADPRT domain.

### Exposure to ExoS/GAP leads to upregulation and mobilization of Bcl-2 pro-apoptotic proteins to the mitochondrial membrane

Mitochondrial outer-membrane stability and/or its permeabilization is largely regulated by Bcl-2 family proteins^43^. These proteins are either anti-apoptotic (i.e. Bcl-2 and Bcl-_XL_), or pro-apoptotic (i.e. Bax, Bak, Bim, and Bid). In healthy cells, Bak, Bim, Bcl-2, and Bcl-_XL_ are subcellularly localized in mitochondria, while Bax and Bid are mainly cytosolic^43,44^. Anti-apoptotic Bcl-2 proteins promote survival by binding Bax and preventing its localization and oligomerization in mitochondrial outer-membrane, thus enhancing mitochondrial integrity^43^. However, when apoptosis is triggered, Bim and Bid become enriched and activated in the mitochondrial outer-membrane where they initiate apoptosis by sequestering Bcl-2 and Bcl-_XL_ pro-survival proteins, liberating Bax to enrich in the mitochondrial outer-membrane, where it forms large complexes by oligomerization that permeabilize the mitochondrial outer-membrane, casing cytochrome *c* to be released into the cytosol^43^.

**Figure 2:**
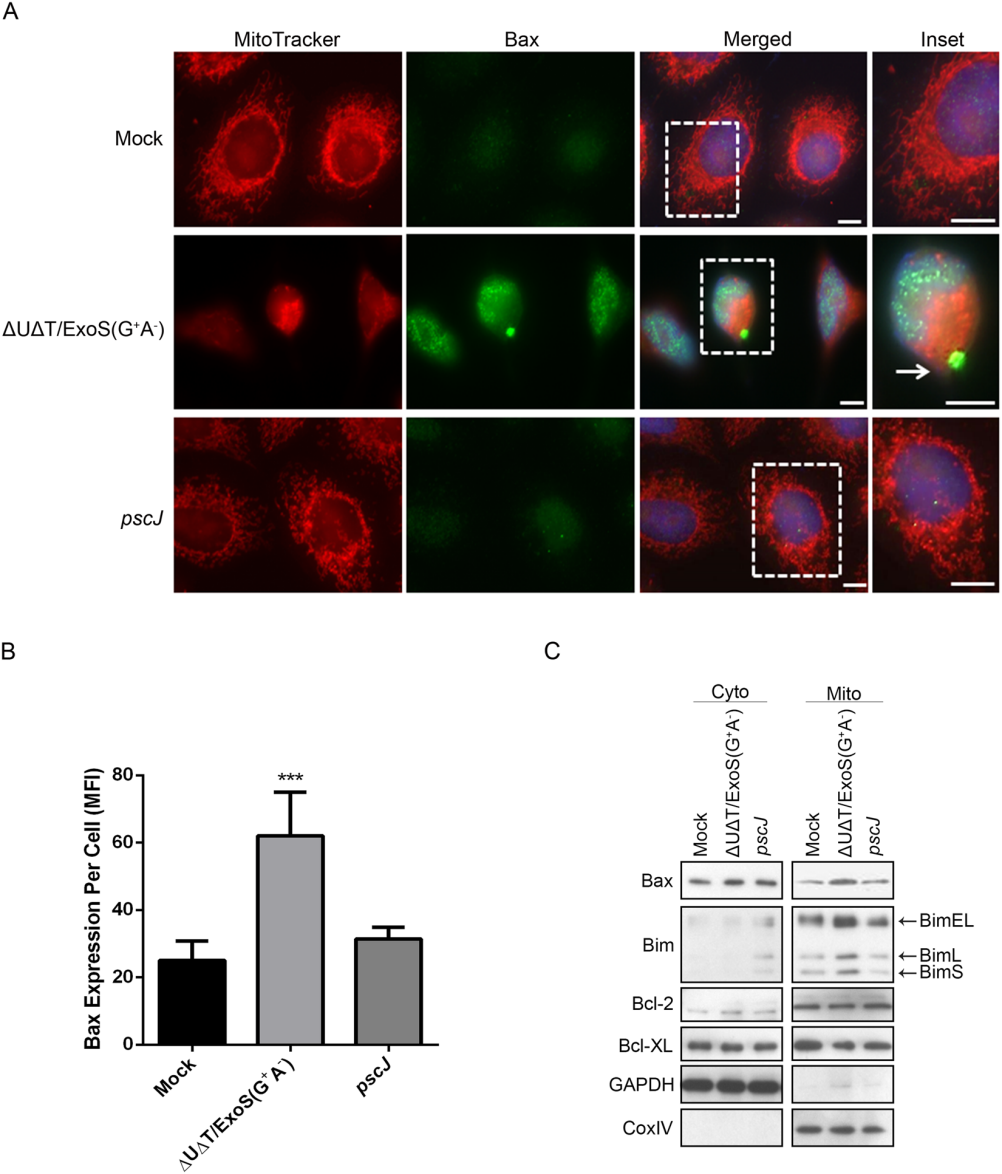
Intoxication with ExoS/GAP results in upregulation and mobilization of Bcl-2 pro-apoptotic proteins to the mitochondrial membrane. HeLa cells were treated with PBS (Mock) or infected with ExoS/GAP-expressing ∆U∆T/ExoS (G^+^A^−^) or the T3SS mutant (*pscJ*) at a MOI of 10. (**A**) 5 h following infection, the cells were fixed and analyzed by IF microscopy after staining for Bax (green), the mitochondrial marker MitoTracker (red), and DAPI nuclear stain (blue). Scale bar represents 20 μm. The arrow points to a Bax globular structure at the mitochondria. (**B**) Bax expression was assessed by determining the mean fluorescent intensity (MFI) from 10 random fields and are presented as the Mean ± SD (***p < 0.001, One-way ANOVA, ns = not significant). (**C**) 5 h following infection, levels of pro-apoptotic proteins Bax and Bim, and anti-apoptotic proteins Bcl-2 and Bcl-_XL_, in cytosolic and mitochondrial fractions were determined by Western blotting. GAPDH and Cox IV were used as loading controls for cytoplasmic and mitochondrial fractions respectively (Equal amounts of proteins were loaded on different gels. The gels were then probed with either Bax, Bim, Bcl-2, Bcl-XL, GAPDH, or Cox IV. Each experiment was repeated at least 3 times and a representative image is shown).

To assess the impact of ExoS/GAP domain on pro- and anti-apoptotic Bcl-2 family proteins, we treated HeLa cells with PBS (Mock) or infected them with GAP-expressing strain (∆U∆T/ExoS (G^+^A^−^)), or the T3SS mutant strain (*pscJ*). We omitted ∆U∆T/ExoS (G^−^A^+^) and ∆U∆T/ExoS (G^−^A^−^) strains from these studies, as they had no significant impact on mitochondrial outer-membrane disruption and cytochrome *c* release into the cytosol (Fig. 1). Five hours post-infection, we fixed the cells and assessed them for Bax by IF microscopy, using the mitochondrial marker MitoTracker (red) and an antibody against Bax (green). As determined by the mean fluorescent intensity (MFI), Bax was significantly upregulated in HeLa cells infected with ExoS/GAP-expressing ∆U∆T/ExoS (G^+^A^−^) strain, as compared to HeLa cells infected with the T3SS mutant strain (*pscJ*) or uninfected HeLa cells (Fig. 2A,B). Interestingly, we also found that in some ∆U∆T/ExoS (G^+^A^−^) infected HeLa cells, Bax formed large globular structures which co-localized with MitoTracker in the mitochondria (Fig. 2A, arrow points to one such structure). Although these Bax globular structures were rare, they were only seen in HeLa cells infected with ∆U∆T/ExoS (G^+^A^−^) and were never seen in HeLa cells infected with the T3SS mutant strain (*pscJ*) or in the uninfected healthy cells, suggesting that Bax oligomerization and mitochondrial membrane disruption may be a rapid event occurring during intrinsic apoptosis.

To corroborate these data, we repeated these infection studies and assessed the impact of ExoS/GAP on Bax and Bim (pro-apoptotic), and Bcl-2 and Bcl-_XL_ (anti-apoptotic) proteins in mitochondrial fractions by Western blotting. Consistent with the data in Fig. 2A,B, infection with ∆U∆T/ExoS (G^+^A^−^) led to increased levels of Bax and the three isoforms of Bim (BimL, BimEL, and BimS, which can all induce apoptosis^45,46^), in the mitochondrial fraction of infected HeLa cells (Fig. 2C). In contrast, ExoS/GAP did not impact Bcl-_XL_ or Bcl-2 levels in the mitochondrial fraction.

### Intoxication with ExoS/GAP domain results in caspase-9 and caspase-3 activation

**Figure 3:**
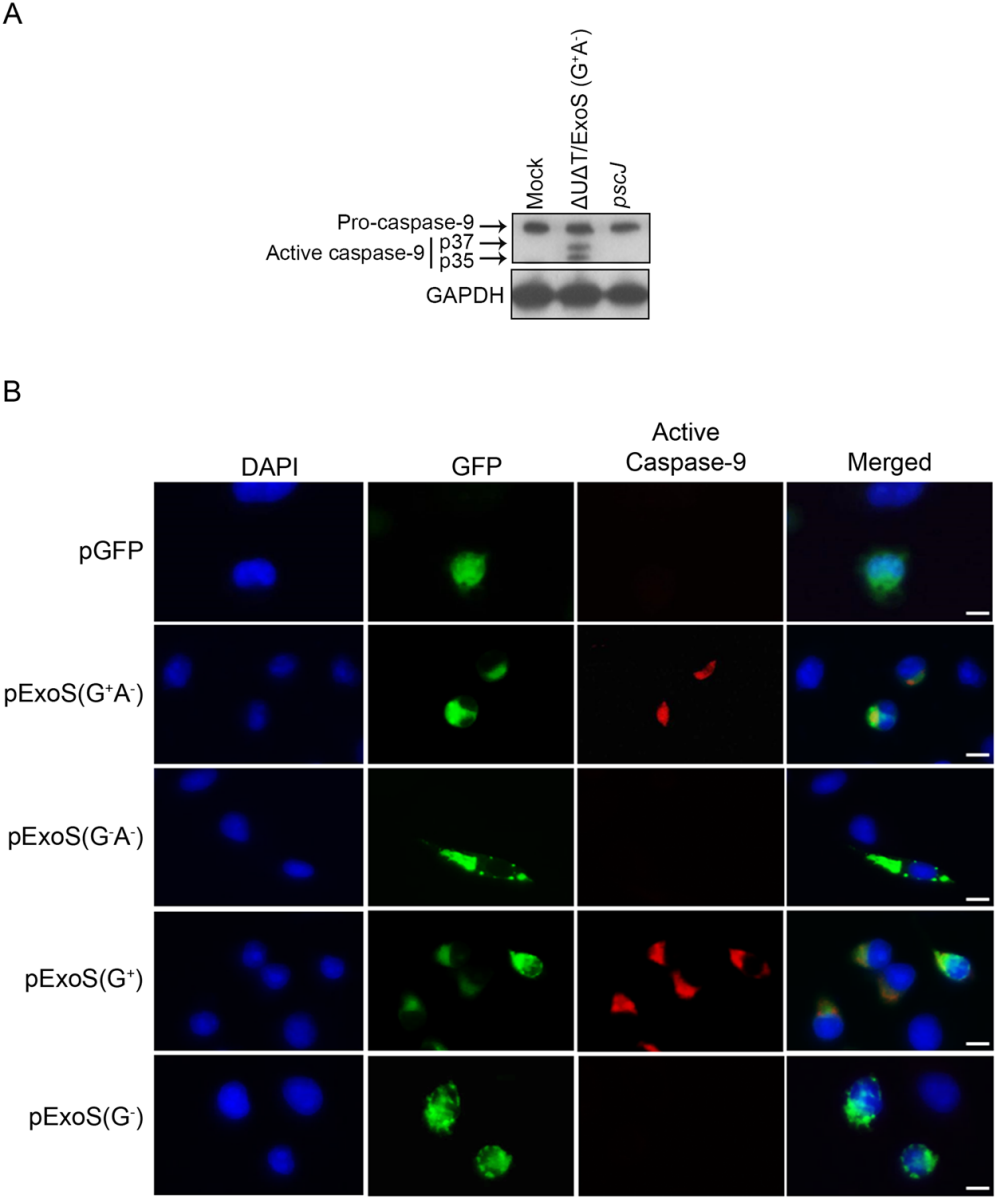
Intoxication with ExoS/GAP domain results in caspase-9 activation. (**A**) HeLa cells were treated with PBS (Mock) or infected with ExoS/GAP-expressing ∆U∆T/ExoS (G^+^A^−^) or the T3SS mutant (*pscJ*) at an MOI of 10. Following a 5 h infection, HeLa cells were harvested and probed for active caspase-9 by Western blotting. GAPDH was used as a loading control (Equal amounts of proteins were loaded on 2 different gels and probed for either caspase-9 or GAPDH. Each experiment was repeated at least 3 times and a representative blot of each is shown). (**B**) HeLa cells were transfected with pIRES2-EGFP mammalian expression control vector (pGFP) or vectors containing full length and truncated GAP-expressing ExoS (pExoS (G^+^A^−^) or pExoS (G^+^)), or their inactive GAP counterparts (pExoS (G^−^A^−^) or pExoS (G^−^)), all directly fused to GFP at their C-termini. 20 h following transfection, cells were fixed and probed for nucleus (blue), gene of interest (GFP), and active caspase-9 (red) by IF microscopy. Note that infection or transfection with functional ExoS/GAP leads to caspase-9 activation. Scale bar represents 20 μm.

Following mitochondrial disruption, cytosolic cytochrome *c* binds to Apaf-1 protein to form the apoptosome, which then recruits and activates the inactive pro-caspase-9 into cleaved active initiator caspase-9, which then cleaves inactive pro-caspase-3 to produce active effector caspase-3^47,48^. To assess the role of ExoS/GAP domain activity in caspase-9 activation, we treated HeLa cells with PBS (Mock) or infected them with ΔUΔT/ExoS (G^+^A^−^) or *pscJ* at a MOI of 10. Following a 5 h infection, HeLa cells were harvested and assessed for caspase-9 activation by Western blotting. Pro-caspase-9 was cleaved into its two active forms (p37 and p35) in cells infected with ΔUΔT/ExoS (G^+^A^−^) but not in untreated cells or cells infected with *pscJ* (Fig. 3A), indicating that intoxication with the GAP domain of ExoS results in caspase-9 activation.

To assess if ExoS/GAP domain was sufficient to activate caspase-9, we transfected HeLa cells with the aforementioned mammalian expression vectors expressing full length or truncated functional ExoS/GAP (pExoS (G^+^A^−^) or pExoS (G^+^)), or full length or truncated inactive GAP (pExoS (G^−^A^−^) or pExoS (G^−^)), or the control vector pGFP. Following a 20 h transfection, we fixed the cells and probed them for active caspase-9 by IF microscopy. Cells that were transfected with pExoS (G^+^A^−^) or pExoS (G^+^) exhibited caspase-9 activation (Fig. 3B). In contrast, caspase-9 was not activated in untransfected cells or cells transfected with pExoS (G^−^A^−^), pExoS (G^−^), or pGFP (Fig. 3B).

**Figure 4:**
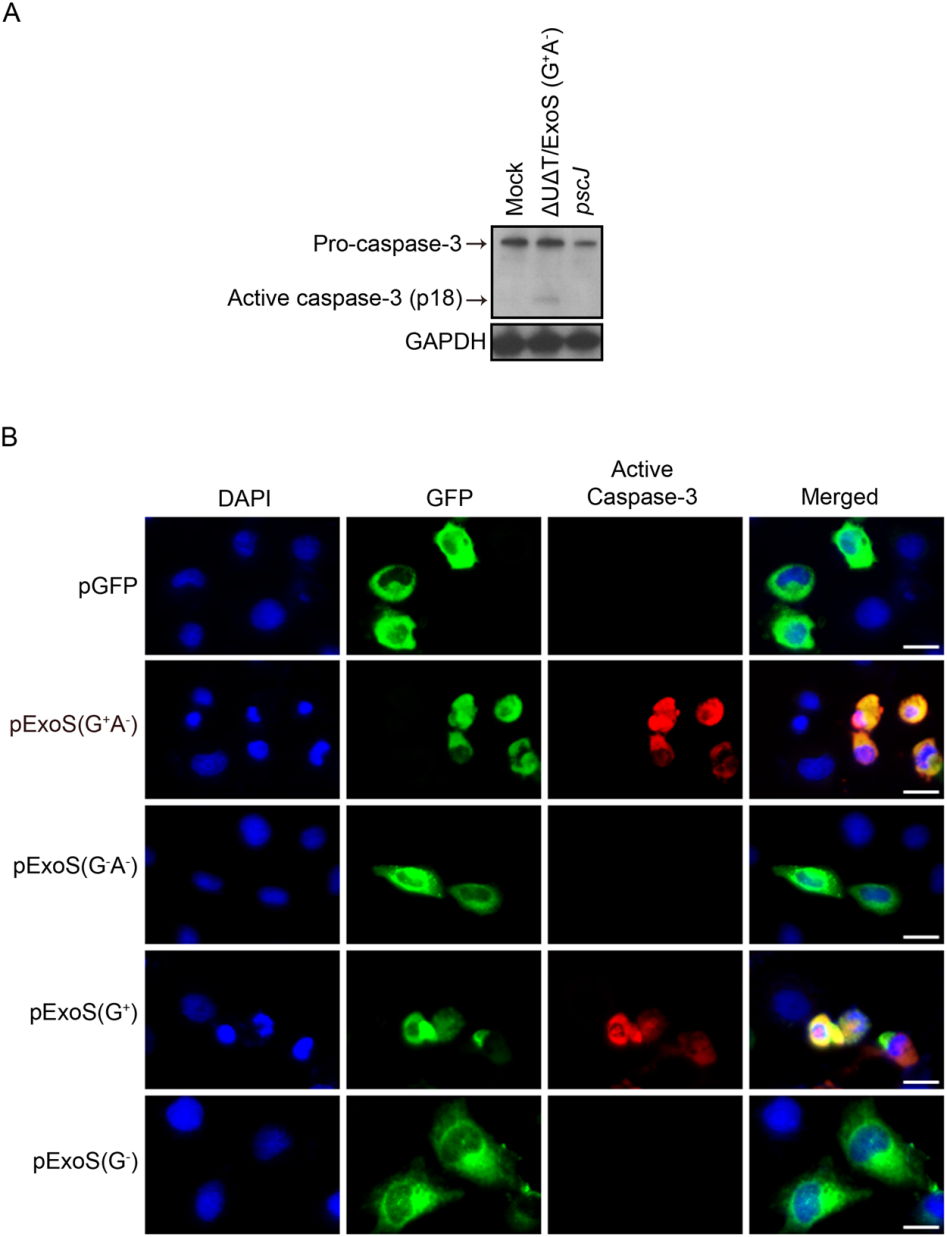
Intoxication with ExoS/GAP domain results in caspase-3 activation. (**A**) HeLa cells were treated with PBS (Mock) or infected with indicated strains as described in Fig. 3 legend. Following a 5 h infection, HeLa cells were harvested and probed for active caspase-3 by Western blotting. GAPDH was used as a loading control. (Equal amounts of proteins were loaded on 2 different gels and probed for either caspase-3 or GAPDH. Each experiment was repeated at least 3 times and a representative blot of each is shown). (**B**) HeLa cells were transfected with pIRES2-EGFP mammalian expression control vector (pGFP) or the vectors containing full length and truncated GAP-expressing ExoS (pExoS (G^+^A^−^) or pExoS (G^+^)), or their inactive GAP counterparts (pExoS (G^−^A^−^) or pExoS (G^−^)), all directly fused to GFP at their c-termini. 20 h following transfection, cells were fixed and probed for nucleus (blue), gene of interest (GFP), and active caspase-3 (red) by IF microscopy. Note that infection or transfection with functional ExoS/GAP leads to caspase-3 activation. Scale bar represents 20 μm.

We conducted similar infection and transfection studies to assess the impact of ExoS/GAP domain activity on caspase-3 activation. Not surprisingly, we found caspase-3 to become activated in cells that were intoxicated with full length or truncated functional GAP domain of ExoS, but not when they were untreated or intoxicated with inactive forms of ExoS/GAP (Fig. 4). Collectively, these data indicated that ExoS/GAP domain is sufficient to activate initiator caspase-9 and executioner caspase-3.

### ExoS/GAP-induced cytotoxicity requires caspase-9 and caspase-3 activities

Mitochondrial outer-membrane permeabilization (MOMP) and caspase activation do not necessarily have to lead to cell death, as these phenomena have also been reported to occur in the context of “non-lethal processes” and for this reason, they are no longer accepted as stand-alone markers for apoptosis according to the Nomenclature Committee on Cell Death (NCCD)^49–52^. Moreover, mitochondrial membrane disruption can also lead to caspase-9 and caspase-3- independent necrotic cell death^43,52–55^. Although our data clearly indicated that caspase-9 and caspase-3 become activated following intoxication with the GAP domain of ExoS (Figs 3 and 4), two questions remained; (1) Does the GAP domain of ExoS have the ability to cause cytotoxicity? (2) Assuming that it does, is ExoS/GAP-induced cytotoxicity apoptotic in nature and dependent on caspase-9 and/or caspase-3 activities?

**Figure 5:**
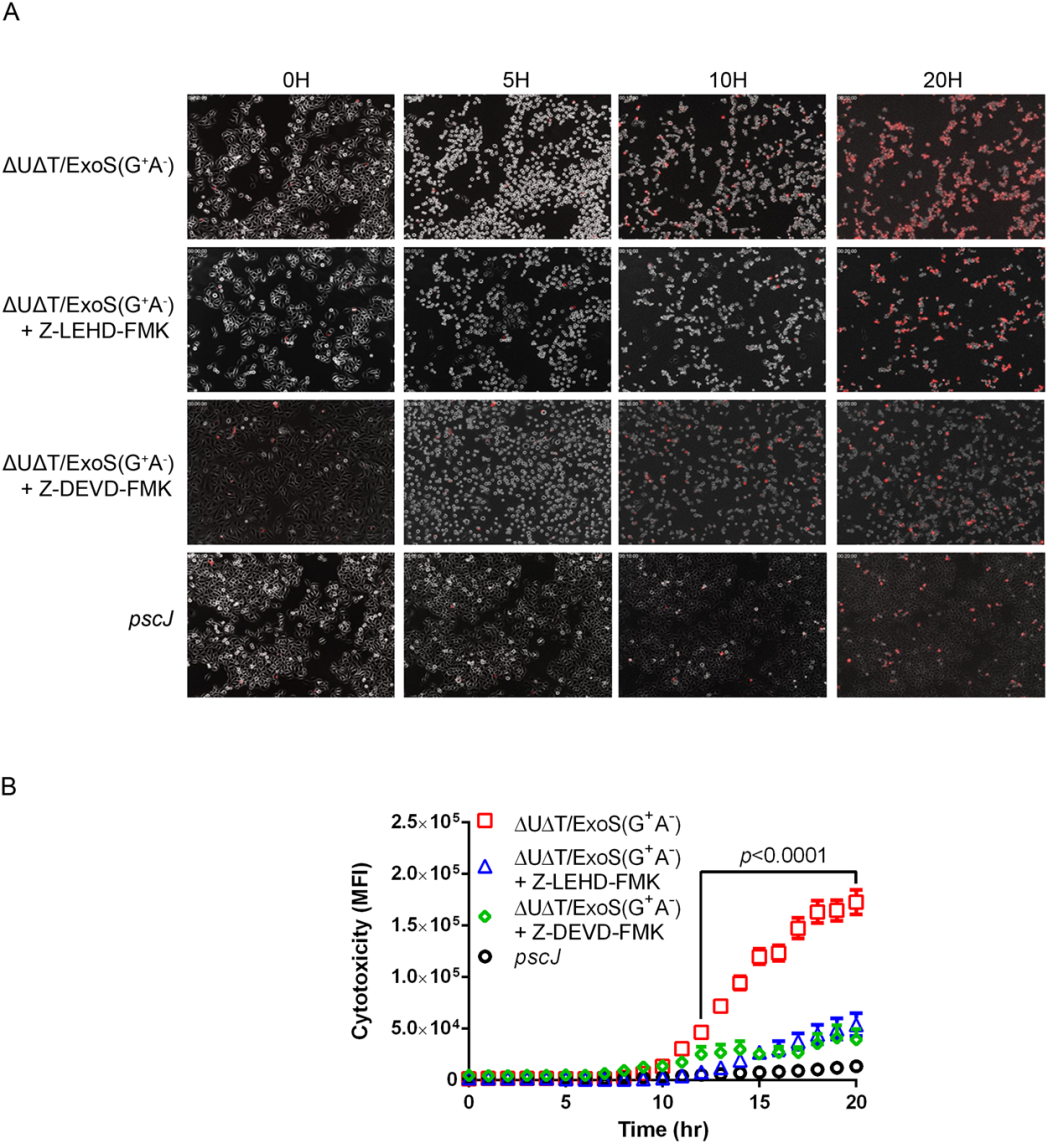
Caspase-9 and caspase-3 activities are required to mediate ExoS/GAP-induced cytotoxicity. HeLa cells were infected with ExoS/GAP-expressing ∆U∆T/ExoS (G^+^A^−^) or the T3SS mutant (*pscJ*) at a MOI of 10 in the absence or presence of caspase-9 inhibitor (Z-LEHD-FMK) or caspase-3 inhibitor (Z-DEVD-FMK). (**A**) Cytotoxicity was assessed by time-lapse videomicroscopy using propidium iodide (PI) as a marker for cell death, as described^12,20^. Images were taken every 15 min for 20 h. Representative images from videos are shown. (**B**) Cytotoxicity was assessed by determining mean fluorescent intensity (MFI) of PI from 3 independent experiments and are presented as the Mean ± SD. Range of significant *p*-values (as determined by One-way ANOVA), are indicated by arrows.

To address these questions, we infected HeLa cells with ∆U∆T/ExoS (G^+^A^−^) or the T3SS defective mutant strain *pscJ*, in the presence or absence of Z-LEHD-FMK (caspase-9 specific inhibitor) or Z-DEVD-FMK (caspase-3 specific inhibitor)^56^. We then assessed cell death by time-lapse videomicroscopy using propidium iodide (PI) uptake, as described previously^12,20,21^ (PI uptake is an acceptable marker for cell death as it indicates the breach in both plasma and nuclear membranes and signifies a point of no return according to NCCD^51,57^). Infection with ExoS/GAP-expressing *P. aeruginosa* strain, but not the T3SS mutant strain, resulted in significant cytotoxicity which began ~12 h after infection and peaked at 18–20 h post-infection (Fig. 5, Movie S1A,B). Importantly, pre-treatment with caspase-9 or caspase-3 specific inhibitors substantially protected against ExoS/GAP-induced cytotoxicity in HeLa cells (Fig. 5, Movie S1C,D), indicating that ExoS/GAP causes intrinsic apoptotic cell death, mediated by the initiator caspase-9 and the effector caspase-3.

**Figure 6:**
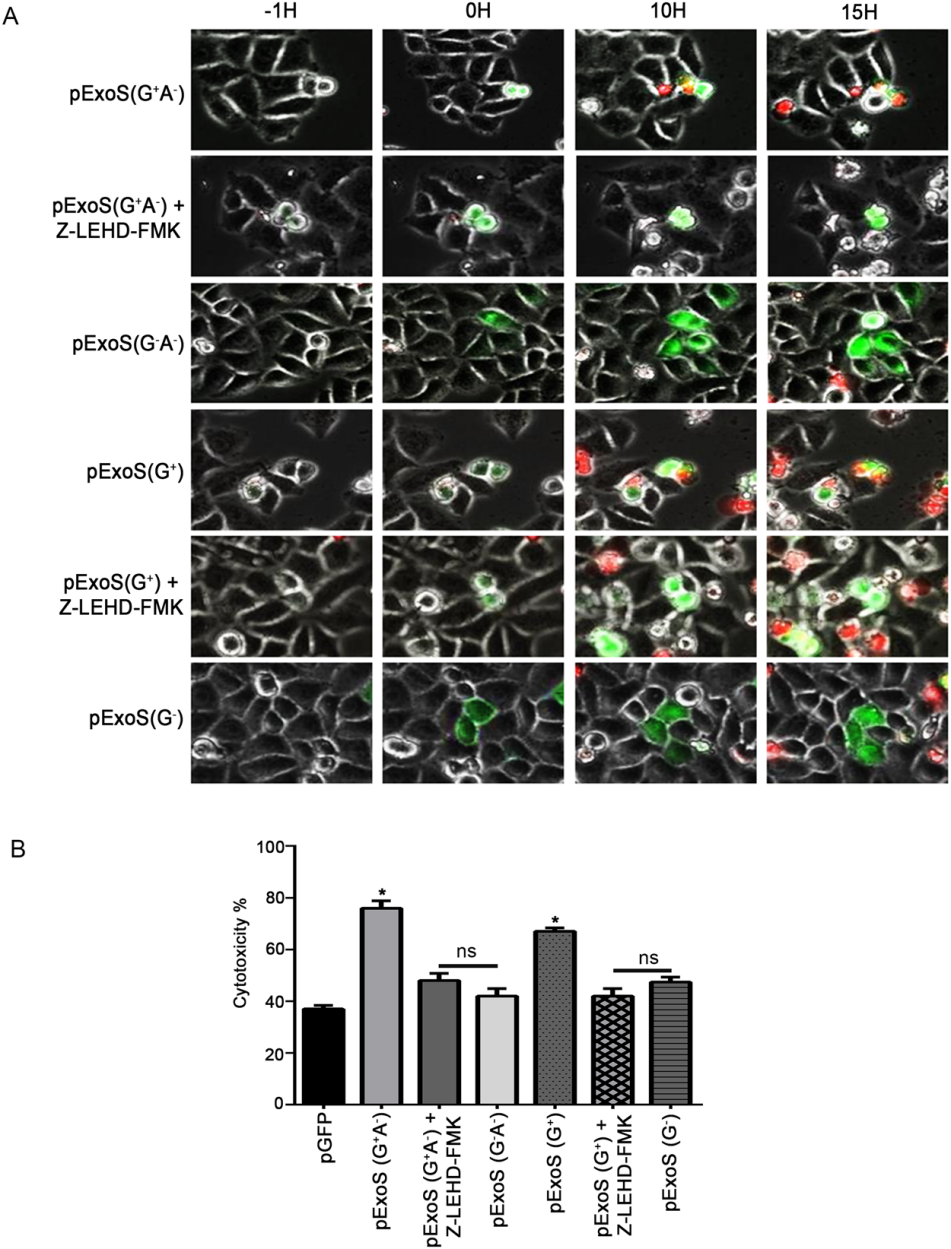
ExoS/GAP domain is sufficient in inducing apoptosis in target host cells. HeLa cells were transfected with pIRES2-EGFP mammalian expression control vector (pGFP) or vectors containing full length and truncated GAP-expressing ExoS (pExoS (G^+^A^−^) or pExoS (G^+^)), or their inactive GAP counterparts (pExoS (G^−^A^−^) or pExoS (G^−^)), all directly fused at their C-termini to GFP. Cytotoxicity was analyzed by IF time-lapse videomicroscopy using PI uptake as a marker for cell death. Images were taken every 15 min. (**A**) Representative images from videos are shown. (−1H) timepoint shows the transfected cells 1 h prior to GFP expression and 0 H is the moment when GFP is expressed. Note that these times are different from the actual timestamps in the movies but were used to normalize data with respect to transfection and cell death to show ExoS/GAP kinetics of cytotoxicity. (**B**) Cytotoxicity was measured as previously described^19^ and the data are presented as the Mean ± SD (ns = not significant, *p < 0.01, Student’s *t*-test).

We next determined if ExoS/GAP domain was sufficient to induce caspase-9 dependent intrinsic apoptosis by transfecting HeLa cells with pExoS (G^+^A^−^), pExoS (G^−^A^−^), pExoS (G^+^), pExoS (G^−^), and pGFP control vector, and assessed cytotoxicity by time-lapse videomicroscopy in the presence or absence of caspase-9 inhibitor Z-LEHD-FMK, as we previously described^21,27^. Transfection with pExoS (G^+^A^−^) or pExoS (G^+^), which expressed functional ExoS/GAP, resulted in significantly more cell death than pExoS (G^−^A^−^), pExoS (G^−^), or pGFP empty vector, which did not contain ExoS/GAP (Fig. 6, Movie S2A–D). Of note, these vectors had similar transfection efficiencies (Fig. S1B). Again, pre-treatment with caspase-9 inhibitor Z-LEHD-FMK significantly reduced apoptosis in HeLa cells transfected with pExoS (G^+^A^−^) or pExoS (G^+^), indicating that ExoS/GAP-induced cytotoxicity is dependent on caspase-9 activity (Fig. 6A,B, Movie S2E,F). These data indicated that ExoS/GAP domain activity is sufficient to cause intrinsic/mitochondrial apoptosis.

### ExoS/GAP-induced cytotoxicity contributes to ExoS-induced cytotoxicity

Prior studies found ExoS-induced apoptosis to be primarly due to its ADPRT domain activity, albeit they primarily had assessed cytotoxicity at earlier timepoints (1–5 h) post-infection^28,29,34^. Although our data indicated that the ExoS/GAP domain activity is capable of causing apoptosis in the absence of the ADPRT domain activity, whether it contributed to ExoS-induced apoptosis in the context of the full-length ExoS protein containing both domains remained unclear.

**Figure 7:**
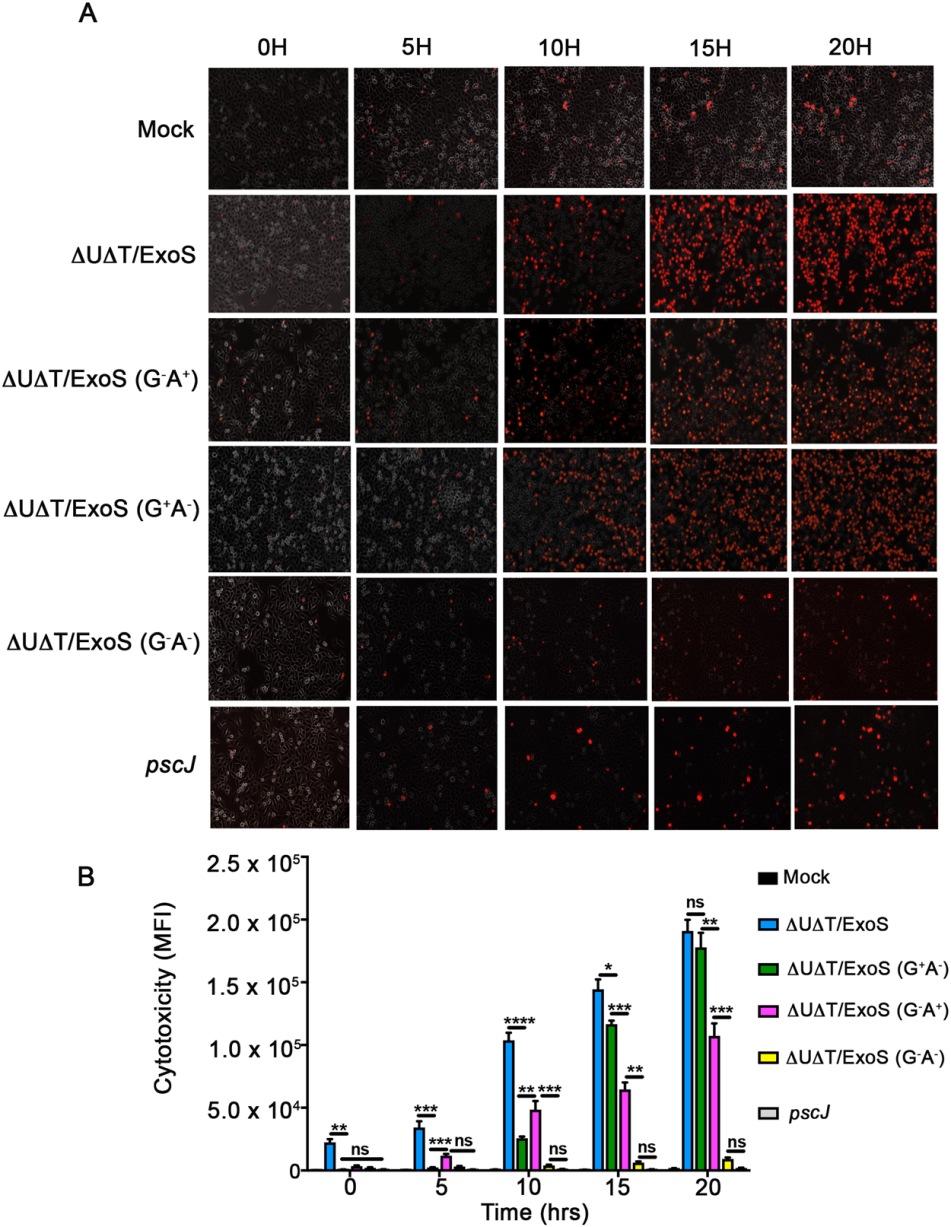
Dynamics of cytotoxicity associated with ExoS and its ADPRT and GAP domains in PA103 strain genetic background. HeLa cells were infected with ExoS-expressing ∆U∆T/ExoS, ExoS/GAP-expressing ∆U∆T/ExoS (G^+^A^−^), ExoS/ADPRT-expressing ∆U∆T/ExoS (G^−^A^+^) strain; or the T3SS mutant (*pscJ*) at a MOI of 10. (**A**) Cytotoxicity was observed by IF time-lapse videomicroscopy using PI uptake as a marker for cell death. Images were taken every 15 min for 20 h. Representative images from videos at indicated times are shown. (**B**) Cytotoxicity was assessed by determining mean fluorescent intensity (MFI) of PI from 3 independent experiments and are presented as the Mean ± SD (****p ≤ 0.0001, ***p ≤ 0.001, **p ≤ 0.01, *p ≤ 0.05, ns = not significant, determined by One-way ANOVA).

To assess the dynamics of ExoS-induced cytotoxicity and to determine the contributions of ADPRT and GAP domain activities in ExoS-induced cytotoxicity, we infected HeLa cells with *P. aeruginosa* strains expressing wildtype ExoS (∆U∆T/ExoS); ExoS with functional GAP and mutant ADPRT (∆U∆T/ExoS (G^+^A^−^)); ExoS with functional ADPRT and mutant GAP (∆U∆T/ExoS (G^−^A^+^)); ExoS with mutant GAP and mutant ADPRT (∆U∆T/ExoS (G^−^A^−^)), or T3SS mutant *pscJ::gent*^*R*^ (*pscJ*), and assessed cytotoxicity by time-lapse videomicroscopy, using propidium iodide (PI) uptake as a marker for cell death. At all timepoints, the level of cytotoxicity associated with ExoS was significantly higher than the cytotoxicity levels associated with either of its domains alone, indicating that ADPRT and GAP domain activities contribute to ExoS-induced cytotoxicity (Fig. 7, Movies S3A–D and S1A). Of note, ExoS-induced cytotoxicity at the 5 h timepoint (the timepoint at which ExoS-induced cytotoxicity was assessed in prior studies^28,29^), only accounted for 17.9% of total ExoS-induced cell death which occurred maximally at the 20 h timepoint after infection (Fig. 7B). Moreover, ExoS/ADPRT-induced cytotoxicity was significantly higher than the GAP-induced cytotoxicity at earlier timepoints within the first 10 h post-infection, while the ExoS/GAP domain activity caused significantly more cytotoxicity than the ADPRT domain at later timepoints (15–20 h post infection) when ExoS-induced cytotoxicity reached its maximum (Fig. 7, Movies S1A,B and S3B). Very little cytotoxicity was observed in cells infected with either the T3SS mutant strain (*pscJ*) or the GAP and ADPRT double mutant strain, ∆U∆T/ExoS (G^−^A^−^) (Fig. 7, Movies S1A and S3C), highlighting the important cytotoxic role of ExoS in this strain. Collectively, these data indicate that both domains of ExoS contribute to ExoS-induced cytotoxicity.

### Impact of bacterial genetic background on ExoS/GAP-induced cytotoxicity

The PA103 surrogate strain used in these studies to assess ExoS/GAP-induced cytotoxicity does not naturally encode the *exoS* gene. To determine the cytotoxic role of ExoS/GAP domain in an ExoS-expressing *P. aeruginosa* strain, we constructed similar strains in the PAK strain genetic background, which naturally expresses ExoS, ExoT, and ExoY T3SS effector proteins, although ExoY plays no role in cytotoxicity induced by this strain^28,29,58^ (See Table S1 and Methods for information regarding these constructed strains). Of note, ExoS-induced apoptosis was originally demonstrated in the PAK strain^28,29^. We infected HeLa cells with PAK strains, expressing wildtype ExoS (∆S∆T/ExoS); ExoS with functional ADPRT and mutant GAP (∆S∆T/ExoS (G^−^A^+^)); ExoS with functional GAP and mutant ADPRT (∆S∆T/ExoS (G^+^A^−^)); ExoS with mutant GAP and mutant ADPRT (∆S∆T/ExoS (G^−^A^−^)); or the T3SS mutant (*pscJ*), and assessed the contributions of the GAP and the ADPRT domains to ExoS-induced mitochondrial membrane disruption and cytochrome *c* release and cytotoxicity in this strain genetic background.

**Figure 8:**
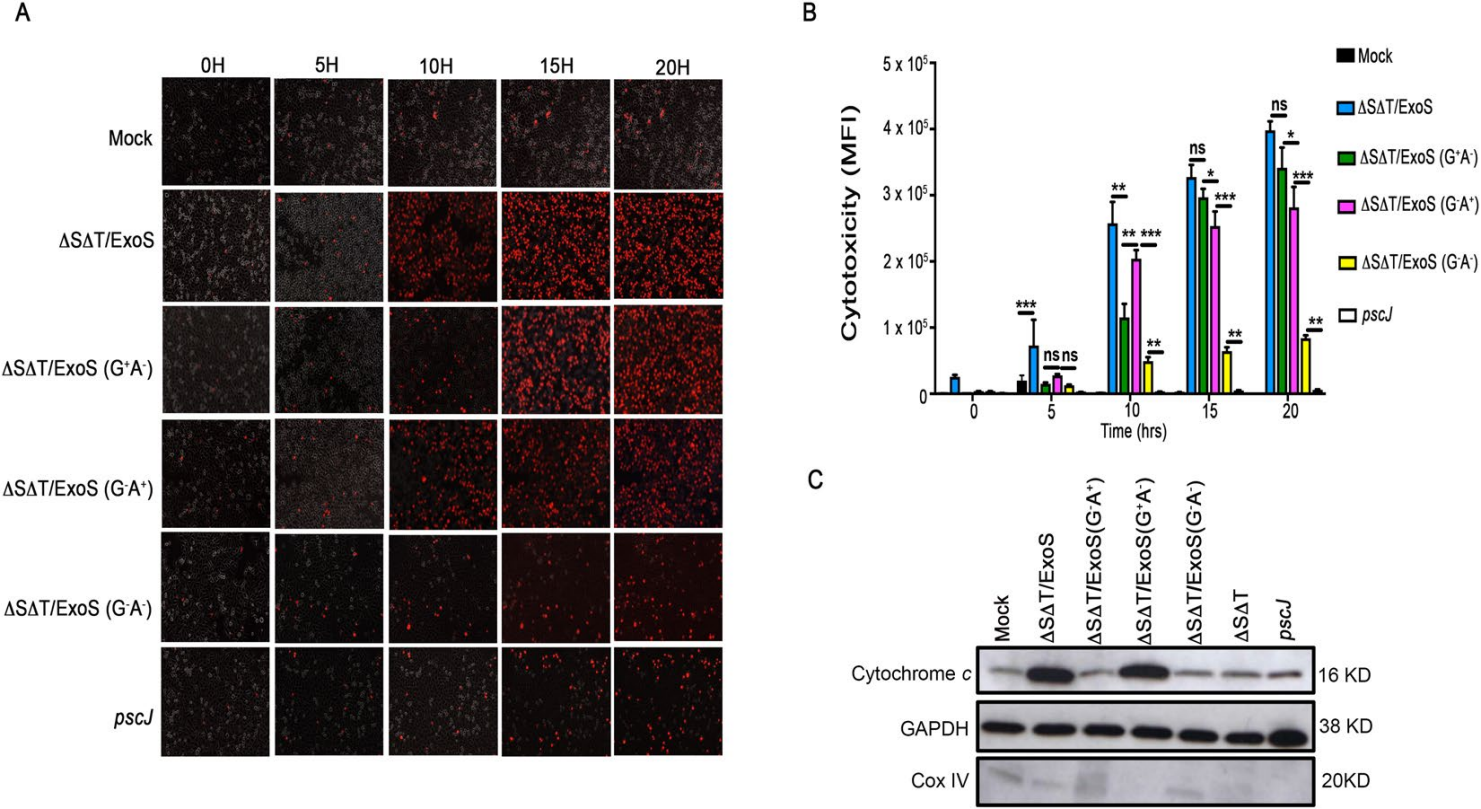
Dynamics of cytotoxicity associated with ExoS and its ADPRT and GAP domains in PAK genetic background. HeLa cells were infected with PAK∆S∆T strains expressing ExoS (∆S∆T/ExoS), ExoS/GAP-expressing ∆S∆T/ExoS (G^+^A^−^), ExoS/ADPRT-expressing ∆S∆T/ExoS (G^−^A^+^) strain; or the T3SS mutant (*pscJ*) at a MOI of 10. (**A**) Cytotoxicity was observed by IF time-lapse videomicroscopy using PI uptake as a marker for cell death. Images were taken every 15 min for 20 h. Representative images from videos are shown (Red cells are dead). (**B**) Cytotoxicity was assessed by determining mean fluorescent intensity (MFI) of PI from 3 independent experiments and are presented as the Mean ± SD (****p < 0.0001, ***p ≤ 0.001, **p ≤ 0.01, *p ≤ 0.05, ns = not significant, determined by by One-way ANOVA). (**C**) 5 h following infection with indicated strains, cells were fractionated and the cytosolic fractions were evaluated for their cytochrome *c* contents by Western blotting using an antibody against cytochrome *c*. GAPDH and Cox IV were used as loading controls for cytoplasmic and mitochondrial fractions respectively (Equal amounts of proteins were loaded on 3 gels and run simultaneously. The gels were then probed with either cytochrome *c*, GAPDH, or Cox IV. Each experiment was repeated at least 3 times and a representative blot of each is shown).

Similar to the data obtained in the PA103 strain genetic background (Fig. 7), cytotoxicity levels associated with ExoS was significantly higher than either of its domains alone in PAK genetic background, indicating that both the ADPRT and the GAP domains contribute to ExoS’s overall cytotoxicity (Fig. 8A,B, Movie S4A–C). Similarly, ExoS-induced cytotoxicity at the 5 h timepoint accounted for only 18.3% of total cytotoxicity associated with ExoS which reached its maximum at 18–20 h post-infection (Fig. 8A,B). Again, ExoS/ADPRT domain was primarily responsible for early cytotoxicity associated with ExoS, while ExoS/GAP domain caused more cytotoxicity at later timepoints (≥15 h) and contributed to overall ExoS-induced cytotoxicity in the PAK genetic background (Fig. 8). Very little cytotoxicity was observed in cells infected with GAP and ADPRT double mutant strain (∆S∆T/ExoS (G^−^A^−^)) or the T3SS mutant *pscJ* strain (Fig. 8, Movie S4D,E), indicating that under these experimental conditions, ExoS-induced cytotoxicity predominates over T3SS-independent cytotoxins. Moreover and consistent with our data in Fig. 1, infection with PAK strains expressing functional GAP (∆S∆T/ExoS and ∆S∆T/ExoS (G^+^A^−^)) resulted in substantial cytochrome *c* release into the cytosol, as assessed by Western blotting (Fig. 8C), indicating that similar to PA103, ExoS/GAP was responsible for mitochondrial disruption and release of cytochrome *c* into the cytosol in PAK genetic background.

## Discussion

In this report, we provide compelling evidence to show that ExoS/GAP induces intrinsic (mitochondrial) apoptosis. Our data demonstrate that ExoS/GAP intoxication results in: (i) upregulation and enrichment of Bax and Bim in mitochondrial outer-membrane, (ii) mitochondrial membrane disruption and cytochrome *c* release into the cytosol, and (iii) caspase-9 and caspase-3 activation, which ultimately drive the ExoS-intoxicated cell toward apoptotic demise. Our data clearly show that the reported ExoS-induced mitochondrial outer-membrane disruption and cytochrome *c* release into the cytosol^29^ is primarily due to ExoS/GAP domain activity and not the ADPRT domain activity.

On the surface, our data seem to contradict previous reports which indicated that ExoS-induced apoptosis is primarily due to its ADPRT domain activity^23,28,29^. These discrepancies could be due to differences in timepoints of analyses, strain genetic backgrounds, and/or target host cells. For example, Kaufman *et al*., Jin *et al*., and Elsen *et al*.^28,29,34^ assessed ExoS-induced cytotoxicities at 1–5 h post-infection and found it to be primarily mediated by its ADPRT domain. We also find ExoS-induced cytotoxicity at early timepoints, prior to 10 h post-infection, to be primarily due to the ADPRT domain activity (Figs 5, 7 and 8). Rather, our data indicate that GAP-induced cytotoxicity begins approximately at 12 h post-infection and peaks around 18–20 h post-infection, which is coincident with the peak of ExoS-induced cytotoxicity (Figs 7 and 8). These later timepoints were not evaluated in prior studies.

Moreover, CHA, PAO1, and PAK strains have been previously used to assess ExoS-induced cytotoxicity^28,31,34^. These strains, however, show stark differences in their ability to cause cytotoxicity. For example, CHA mucoid strain has been shown to be substantially more cytotoxic than PAO1 or PAK^34^. Of note, early cytotoxicity induced by CHA is primarily necrotic in nature and apoptosis only accounts for ~5% cell death by this strain^34^. PAO1 also behaves differently than PAK in regard to cytotoxicity. In PAO1 genetic background, ExoT plays a more significant role than ExoS in inducing apoptosis^31^, whereas in PAK strain, ExoT does not play a role in inducing apoptosis^28^. Finally, in these previous studies, HL60 pro-myeloid cell lines (differentiated into macrophages), macrophages, neutrophils, and HeLa epithelial cells were used to assess ExoS-induced cytotoxicity. These cell lines could have different susceptibility to the GAP and/or the ADPRT-induced cytotoxicities. For example, it has been reported that the T3SS is not required for PAO1 strain to survive in macrophages, but it is absolutely essential for this strain to survive in neutrophils^31^.

The mechanism underlying ExoS/GAP-induced intrinisic apoptosis remains to be determined. We posit that ExoS/GAP-induced intrinsic apoptosis likely occurs as a consequence of ExoS inhibitory effect on RhoA small GTPase which is mediated by its GAP domain activity^24,59^. RhoA has been shown to promote survival by stabilizing mitochondrial integrity in various biological systems^60–64^. RhoA blocks apoptosis in T cells by upregulating the expression of the anti-apoptotic protein Bcl-2 in these cells^61^. RhoA protects glomerular epithelial cells from apoptosis by activating extracellular signal-regulated kinase (ERK) survival pathway which also stabilizes mitochondrial membrane integrity^62^. RhoA has been shown to increase the expression of Erk/Bcl-2 survival pathway in zebra fish^65^. RhoA has also been demonstrated to inhibit hypoxia-induced apoptosis and mitochondrial dysfunction in chondrocytes by upregulating CREB phosphorylation^65^. Thus, inhibition of RhoA by ExoS/GAP domain activity^24,59^ would be expected to destabilize mitochondrial membrane and result in apoptosis, as we demonstrate in this report.

We also confirm in this report that the ADPRT domain of ExoS also contributes to ExoS-induced apoptosis both early and at later timepoints, but the mechanism(s) underlying ExoS/ADPRT-induced cell death remains unknown. There are several possible mechanisms that could account for ExoS/ADPRT-induced apoptosis as ExoS/ADPRT domain targets many cellular proteins such as Ras, Rab, and Rel^66–69^, which play crucial roles in cellular survial^70–76^. For example, Ras is an important survival protein that promotes survival in many cell types and systems by activating the phosphatidylinositol 3-kinase PI3K/Akt survival signaling pathway or the Ras/ERK survival signaling pathway^70–73^. Similarly, inhibition of Rel/NF-kB survival function by TGFβ1 leads to reduction of c-*myc* expression and induces apoptosis^75^. Additionally, inhibition of Rab prenylation by phosphonocarboxylate analogue of risedronate has been shown to result in apoptosis in *Caenorhabditis elegans* and in human myeloma cells^74,77^. These possibilities need to be further investigated to determine the mechanism(s) underlying ExoS/ADPRT-induced cytotoxicity.

What is the physiological relevance of ExoS possessing two domains, both capable of inducing cytotoxicity? We propose that during infection, either in a human host or in another environmental host, *P. aeruginosa* may encounter cell types that may be resistant to ExoS/GAP or ExoS/ADPRT-induced cytotoxicity and in that situation, possession of the alternative domain may serve an important physiological role for this pathogen to overcome the host’s survival mechanism. For example, cells can develop resistance to ExoS/ADPRT by reducing the expression of FAS, a 14-3-3 adaptor protein which is required for the ADPRT domain activation in target host cells^8,26^. Under such a scenario, ExoS/GAP domain activity may be crucial in inducing apoptosis in these host cells.

In summary, we provide compelling evidence to show that ExoS/GAP domain is necessary and sufficient to cause intrinsic (mitochondrial) apoptosis. Further, our data clarify the field with respect to the unappreciated function of the GAP domain as a major contibutor to ExoS-induced apoptosis.

## Methods

### Cell Culture and Reagents

HeLa (human epithelial cervical cancer) cells were originally obtained from the American Type Culture Collection (ATCC), as described previously^18^. HeLa cells were grown in complete DMEM (Life Technologies) augmented with phenol red, 10% FBS (Fetal Bovine Serum), 1% L-glutamine, and 1% penicillin/streptomycin at 37 °C with 5% CO_2_. Cells were detached using 0.12% Trypsin (Gibco). For infection studies, HeLa cells were grown in complete DMEM without antibiotics for a period of 5 or 20 hours. For transfection studies, cells were grown in complete DMEM with antibiotics for a period of 20 or 48 hours. For cytotoxicity measurments by immunofluorescent (IF) time-lapse videomicroscopy, cells were grown in media without phenol red.

### Bacterial Preparation

For a complete list of bacteria, plasmids, and their sources, used in these studies refer to Table S1. All strains were either in PA103 or PAK genetic backgrounds and were previously described^2,21,27–29,31^–except PA103ΔUΔT/ExoS, PA103ΔUΔT/ExoS (G^+^A^−^), PA103ΔUΔT/ExoS (G^−^A^+^), PA103ΔUΔT/ExoS (G^−^A^−^), PAK∆S∆T/ExoS, PAKΔSΔT/ExoS (G^+^A^−^), PAKΔSΔT/ExoS (G^−^A^+^), PAKΔSΔT/ExoS (G^−^A^−^) strains, which were constructed for these studies. To construct these strains, pUCP18::ExoS, pUCP18::ExoS (G^+^A^−^), pUCP18::ExoS (G^−^A^+^), and pUCP18::ExoS (G^−^A^−^) plasmids were transformed into PA103ΔUΔT or PAKΔSΔT, as previously described^78^. Briefly, these plasmid constructs were extracted from *Escherichia coli* surrogate strains (generous gift from Dr. Suzanne Fleiszig^79^), using plasmid miniprep following manufacturer’s protocol (Qiagen). 100 ng of the aforementioned plasmids were then transformed into 500 μL bacteria (washed 3x with 300 mM sucrose and resuspended in the same medium) by electroporation in a 0.4 cm gap cuvette (settings: 200 Ω, 25 μF, 2.5 kV). After electroporation, bacteria were resuspended in 1 mL S.O.C. medium and grown in shaking incubator for 1 h at 37 °C. Bacteria were then plated on LB plates containing 200 µg/mL carbenicillin and grown overnight at 37 °C to select for transformants. For infection studies, indicated bacterial strains were cultured in Luria-Bertani (LB) broth containing carbenicillin overnight at 37 °C. Bacteria titers were adjusted to OD_600_ = 0.05 prior to infection and added to HeLa cells at a multiplicity of infection (MOI) of 10.

### Cytotoxicity Assessment by IF Time-lapse Videomicroscopy

Cytotoxicity measurements by time-lapse IF videomicroscopy were performed as previously described^12,21^. Briefly, HeLa cells were seeded at a density of 6 × 10^5^ cells per well overnight at 37 °C with 5% CO_2_. Cells were treated in the absence or presence of 60 μM Z-LEHD-FMK (caspase-9 inhibitor) or 60 μM Z-DEVD-FMK (caspase-3 inhibitor) which were obtained from R&D systems. Cytotoxicity was assessed at 15 min intervals by immunofluorescent (IF) microscopy using propidium iodide (PI) uptake measurements by ImageJ software, as previously described^12,20,21^. PI was added at 7 μg/mL prior to time-lapse videomicroscopy.

### Western Blot

Western blotting was performed as described previously^80,81^. Cells were seeded at a density of 5 × 10^5^ cells per well overnight at 37 °C with 5% CO_2_. Antibodies used in Western blot experiments include Bim (Cell Signaling Technology; 2933), Bax (Cell Signaling Technology; 2774), Bcl-_XL_ (Cell Signaling Technology; 2764), Bcl-2 (Cell Signaling Technology; 2876), GFP (Santa Cruz Biotechnologies; 8334), GAPDH (GenScript; A00191), CoxIV (Cell Signaling Technology; 11967), Caspase-3 (Cell Signaling Technology; 9668), and Caspase-9 (Cell Signaling Technology; 9508). Secondary antibodies used were HRP-linked Anti-Rabbit (Cell Signaling Technology; 7074), Anti-Mouse (Cell Signaling Technology; 7076), and Anti-Goat (Thermo Fisher; A16148). Primary and secondary antibody dilutions were 1:500–1:1000.

### Immunofluorescent Microscopy

Immunofluorescent microscopy was performed as previously described^12,20,21,27^. Coverslips were treated with 1M HCl for 10 min, followed by treatment with poly-l-lysine (Sigma) for 5 min, followed by treatment with 40 μg/ml human fibronectin (Millipore) for 1 h. Following each treatment, coverslips were rinsed with PBS. Cells were then seeded on these coverslips at a density of 6 × 10^5^ cells per well overnight at 37 °C with 5% CO_2_. For experiments using MitoTracker (Thermo Fisher), cells were stained 45 min prior to fixation. Cells were fixed with 10% TCA (Sigma) for 10 min, then permeabilized with 0.2% Triton X-100 (Sigma) for 15 min prior to staining with primary antibody. Primary antibodies used for immunofluorescent experiments include Bax (Abcam; ab5714), cytochrome *c* (Cell Signaling Technology; 12959), cleaved caspase-9 (Cell Signaling Technology; 9505) and cleaved caspase-3 (Cell Signaling Technology; 9661). Secondary antibodies used were Alexa Fluor 594 (Life Technologies, A-11012) and Alexa Fluor 488 (Life Technologies, A-11018). Primary antibody dilution was 1:100–1:200 and secondary antibody dilution was 1:500–1:1000. Coverslips were mounted on slides with DAPI, to stain for nuclei. For mitochondrial health analyses, images were captured by Zeiss LSM 700 confocal microscope. Mean fluorescent intensity (MFI) was measured using ImageJ software.

### Construction of Expression Vectors for Transient Transfection

Mammalian expression vectors were constructed using pIRES2-EGFP containing ExoS (G^+^A^−^), ExoS (G^−^A^−^), ExoS (G^+^), and ExoS (G^−^) were directly fused at their C-termini to GFP. Primer sequences in lowercase indicate engineered restriction sites. ExoS (G^+^A^−^), ExoS (G^−^A^−^), ExoS (G^+^), and ExoS (G^−^) were amplified by PCR as previously described^82,83^ using primers containing *Nhe*1 and *Spe*1 restriction sites; ExoS-F1-*Nhe*1 (5′-ggggaagctagcATGCATATTCAATCGCTTCAGC-3′) and ExoS-R1-*Spe*1 (5′-aaaactagtGGCCAGATCAAGGCC-3′). The mammalian vector pIRES2-EGFP (Clontech) was modified using primers containing *Nhe*1 and *Spe*1 restriction sites; EGFP-r2-*Nhe*1 (5′-aaaagctagcGGATCTGACGGTTCAC-3′) and EGFP-f3-*Spe*1 (5′-aaaactagtATGGTGAGCAAGGGCGAGG-3′). The amplified ExoS and pIRES2-EGFP PCR products were then digested using 5 units each of *Nhe*1 and *Spe*1 overnight at 37 °C. Following digestion, ExoS PCR products were ligated into pIRES2-EGFP using 50 ng vector, 37.5 ng insert, and 2 μl T4 DNA Ligase, following manufacturer’s protocol (New England Biolabs) to create our constructs for transfection, pIRES2::ExoS (G^+^A^−^)-GFP, will be referred to as pExoS (G^+^A^−^); pIRES2::ExoS (G^−^A^−^)-GFP, will be referred to as pExoS (G^−^A^−^); pIRES2::ExoS (G^+^)-GFP, will be referred to as pExoS (G^+^); and pIRES2::ExoS (G^−^)-GFP, will be referred to as pExoS (G^−^). These constructs were confirmed by sequencing.

### Transient Transfection

Transient transfection was performed as previously described^21^. Briefly, HeLa cells were seeded at a density of 4 × 10^4^ cells per well overnight at 37 °C with 5% CO_2_. Effectene transfection reagent was used following manufacturer’s protocol (Qiagen, CA) with 0.4 μg plasmid DNA as previously described^80,81^.

### Quantification of mitochondrial number in cell by Columbus software (Perkin Elmer Inc.)

Mitochondrial numbers were analyzed and quantified using Columbus software (PerkinElmer Inc.) as previously described^84^. For the analysis and quantification of mitochondria number per cell, cell images were imported to Columbus software. Using Columbus, nuclei were initially found by “Find Nuclei” (Method C) with DAPI signal, and then nuclei showing detectable DAPI signal were selected for further analysis. Cytoplasm was found by “Find Cytoplasm” (Method D) with cytochrome *c* signal. Cells that could not be visualized in their entirety in each field were excluded from quantification. Method A of “Find Spots” detected globular spots of cytochrome *c* signal, and the spot numbers per cell were quantified by the software. For transfection experiments with GFP-expressing plasmids, GFP signal intensity in each cell was measured by Columbus software as well. Cells showing threshold levels of GFP signal were selected as GFP-positive (or transfected) cells and the other cells were selected as GFP-negative cells. Cytochrome *c*- positive globular spots per cell in GFP-positive or GFP-negative cells were quantified separately.

### Statistical Analysis

Statistical analyses were performed using GraphPad Prism 6.0 software as described previously^21,80^. Comparison between two groups was performed using Student’s *t*-test. Comparison between more than two groups was performed using One-way ANOVA. To account for error inflation due to multiple testing, the Bonferroni method was used. Statistical significance threshold was set at *p* ≤ 0.05.

## Electronic supplementary material

Supplementary Information

Movie S1. ExoS/GAP domain activity causes cytotoxicity in HeLa cells.

Movie S2. ExoS/GAP domain is sufficient to cause cytotoxicity in HeLa cells.

Movie S3. Dynamics of ExoS/GAP-induced cytotoxicity in PA103 genetic background.

Movie S4. Dynamics of ExoS-induced cytotoxicity in PAK genetic background.
