# Supplementary material for: Pseudomonas aeruginosa ExoS Induces Intrinsic Apoptosis in Target Host Cells in a Manner That is Dependent on its GAP Domain Activity
Source: Sci Rep. 2018 Sep 19;8:14047. doi: 10.1038/s41598-018-32491-2 (PMC6145893; doi:10.1038/s41598-018-32491-2)
Supplement: Supplementary file 1 — Supplementary Information [file 41598_2018_32491_MOESM1_ESM.pdf]

SUPPLEMENTARY INFORMATION

TITLE: *Pseudomonas aeruginosa* ExoS Induces Intrinsic Apoptosis in Target Host Cells in a Manner That is Dependent on its GAP Domain Activity.

AUTHORS: Amber Kaminski[1,2§], Kajal H. Gupta[1,2§], Josef W. Goldufsky[1,2], Ha Won Lee[1], Vineet Gupta[1], and Sasha H. Shafikhani[1,2,3*]

Table 1: Strains and plasmids used in these studies.

Strains and Plasmids	Abbreviated Name	Characteristics	Source or Reference
**Strains**			
PA103 Δ*exoU*, Δ*exoT*	PA103 ΔUΔT	This strain contains in-frame deletion in *exoU* and *exoT* genes	[11,21]
PA103 *pscJ*::*gent*^{R}	PA103 *pscJ*	PA103 with Tn5 Gent^r transposon inserted into *pscJ*, creating defective T3SS	[11,21]
PA103 ΔUΔT+ pUCP18::*exoS*	PA103 ΔUΔT/ExoS	PA103ΔUΔT strain expressing wild-type ExoS	This study
PA103 ΔUΔT+ pUCP18::*exoS*(R146K)	PA103 ΔUΔT/ExoS(G$^-$A$^+$)	PA103ΔUΔT strain expressing ExoS with functional ADPRT and mutated GAP	This study

PA103 ∆U∆T+ pUCP18::*exoS*(E379D,E381D)	PA103 ∆U∆T/ExoS(G^+A^-)	PA103∆U∆T strain expressing ExoS with functional GAP and mutated ADPRT	This study
PA103 ∆U∆T+ pUCP18::*exoS*(R146K, E379D,E381D)	PA103 ∆U∆T/ExoS(G^-A^-)	PA103∆U∆T strain expressing ExoS with mutated GAP and mutated ADPRT	This study
PAK ∆*exoS*, ∆*exoT*	PAK∆S∆T	This strain contains in-frame deletions in *exoS* and *exoT* genes	[28]
PAK *pscJ*::*gent*^{R}	PAK *pscJ*	PAK with Tn5 Gent^r transposon inserted into *pscJ*, creating defective T3SS	[28]
PAK ∆S∆T + pUCP18::*exoS*	PAK ∆S∆T/ExoS	PAK∆S∆T strain expressing wild-type ExoS	This study

PAK ∆S∆T + pUCP18::*exoS*(R146K)	PAK ∆S∆T/ExoS(G^-A^+)	PAK∆S∆T strain expressing ExoS with functional ADPRT and mutated GAP	This study
PAK ∆S∆T + pUCP18::*exoS*(E379D,E381D)	PAK ∆S∆T/ExoS(G^+A^-)	PAK∆S∆T strain expressing ExoS with functional GAP and mutated ADPRT	This study
PAK ∆S∆T + pUCP18::*exoS*(R146K, E379D,E381D)	PAK ∆S∆T/ExoS(G^-A^-)	PAK∆U∆T strain expressing ExoS with mutated GAP and mutated ADPRT	This study
**Plasmids**			
pUCP18::*exoS*(E379D,E381D)	ExoS(G^+A^-)	Contains full length *exoS* with functional GAP & mutant ADPRT	85
pUCP18::*exoS*(R146K, E379D,E381D)	ExoS(G^-A^-)	Contains full length inactive *exoS* with mutant	85

		GAP & mutant ADPRT	
pIRES2-EGFP	pGFP	pIRES2-EGFP empty vector	[27,81]
pIRES2::ExoS(G^+A^-)-GFP	pExoS(G^+A^-)	pIRES2-EGFP harboring full length *exoS* with a functional GAP domain and a mutated ADPRT directly fused at its C-terminus to GFP	This study
pIRES2::ExoS(G^-A^-)-GFP	pExoS(G^-A^-)	pIRES2-EGFP harboring full length *exoS* with GAP and ADPRT double mutant directly fused at its C-terminus to GFP	This study
pIRES2::ExoS(G^+)-GFP	pExoS(G^+)	pIRES2-EGFP harboring truncated *exoS* with functional	This study

		GAP domain directly fused at its C-terminus to GFP	
pIRES2::ExoS(G$^-$)-GFP	pExoS(G$^-$)	pIRES2-EGFP harboring truncated *exoS* with mutated GAP domain directly fused at its C-terminus to GFP	This study

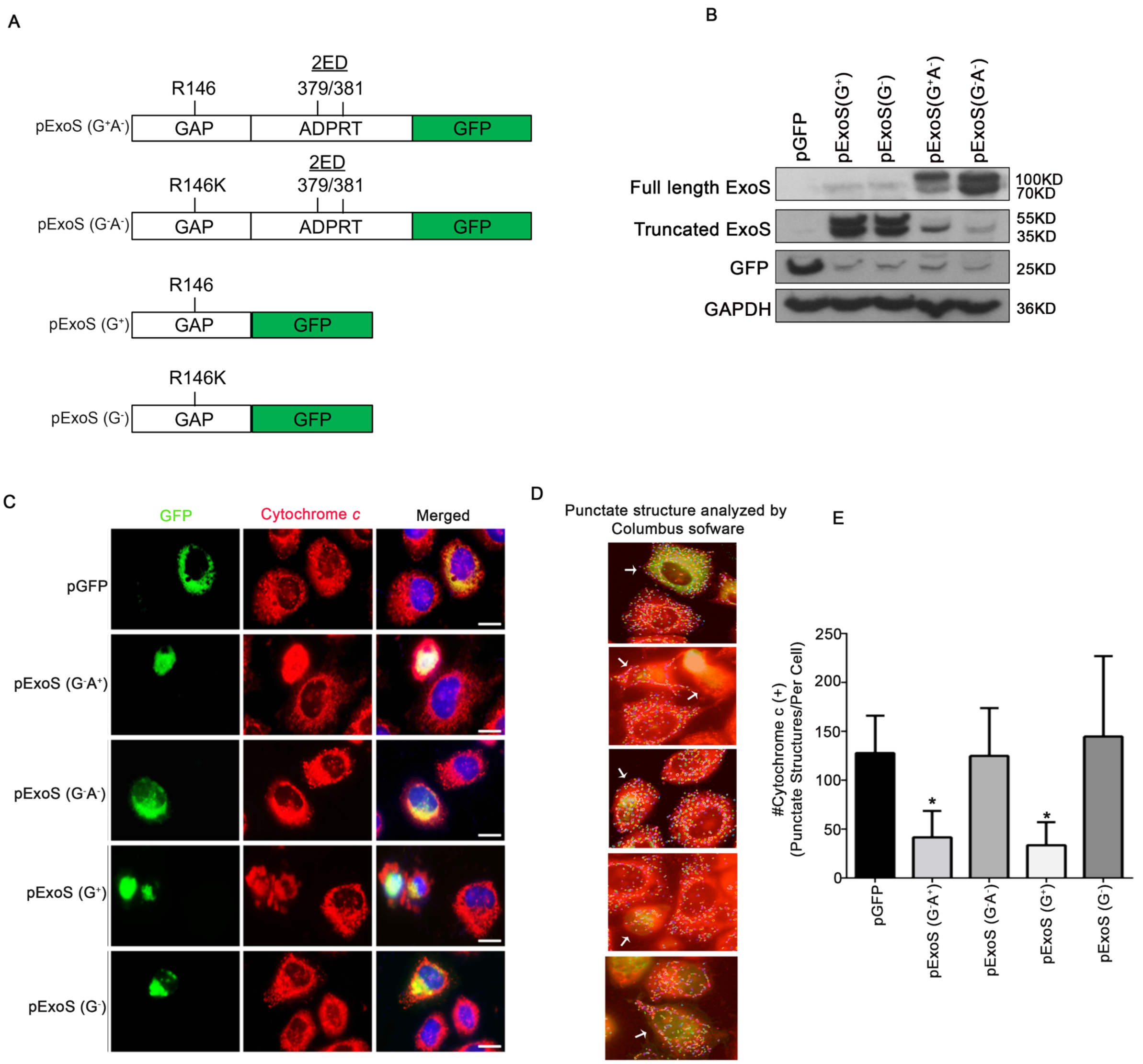

Figure S1. ExoS/GAP domain is sufficient to disrupt mitochondrial membrane and cause cytochrome *c* release into the cytosol. (A) Schematic diagram of full length or truncated ExoS (ExoS (G^{+}A^{-}) or ExoS (G^{+})), or their inactive GAP counterparts (ExoS (G^{-}A^{-}) or ExoS (G^{-})), all directly fused to GFP at their C-termini is shown. **(B-E)** HeLa cells were transfected with pIRES2-EGFP mammalian expression control vector (pGFP) or pIRES2 vectors containing full length or truncated GAP-expressing ExoS (pExoS (G^{+}A^{-}) or pExoS (G^{+})), or their inactive GAP counterparts (pExoS (G^{-}A^{-}) or pExoS (G^{-})), all directly fused to GFP at their C-termini. 20h after transfection, cells were fixed and stained for GFP (green), cytochrome *c* (red), and DAPI nuclear dye (blue), and the impact of ExoS/GAP on mitochondrial health was assessed by IF microscopy. **(B)** Transfection efficiencies of indicated expression vectors were assessed by Western blot, probing with anti-GFP antibody. GAPDH was used as loading control (GAPDH Western was from a different gel but equal amounts were loaded, whereas the GFP Western was from the same gel). **(C)** Representative images of cytochrome *c* stained transfected cells are shown. **(D-E)** Columbus software was used to outline and determine the number of intact mitochondria per cell (cytochrome *c* positive punctate structures represented by different colored circles). Representative Columbus images are shown in (**D**) and the tabulated data, as determined by Columbus software from 10 random fields/groups, are shown as the Mean ± SD in (**E**) (*p < 0.01, one-way ANOVA). Scale bar represents 20μm.

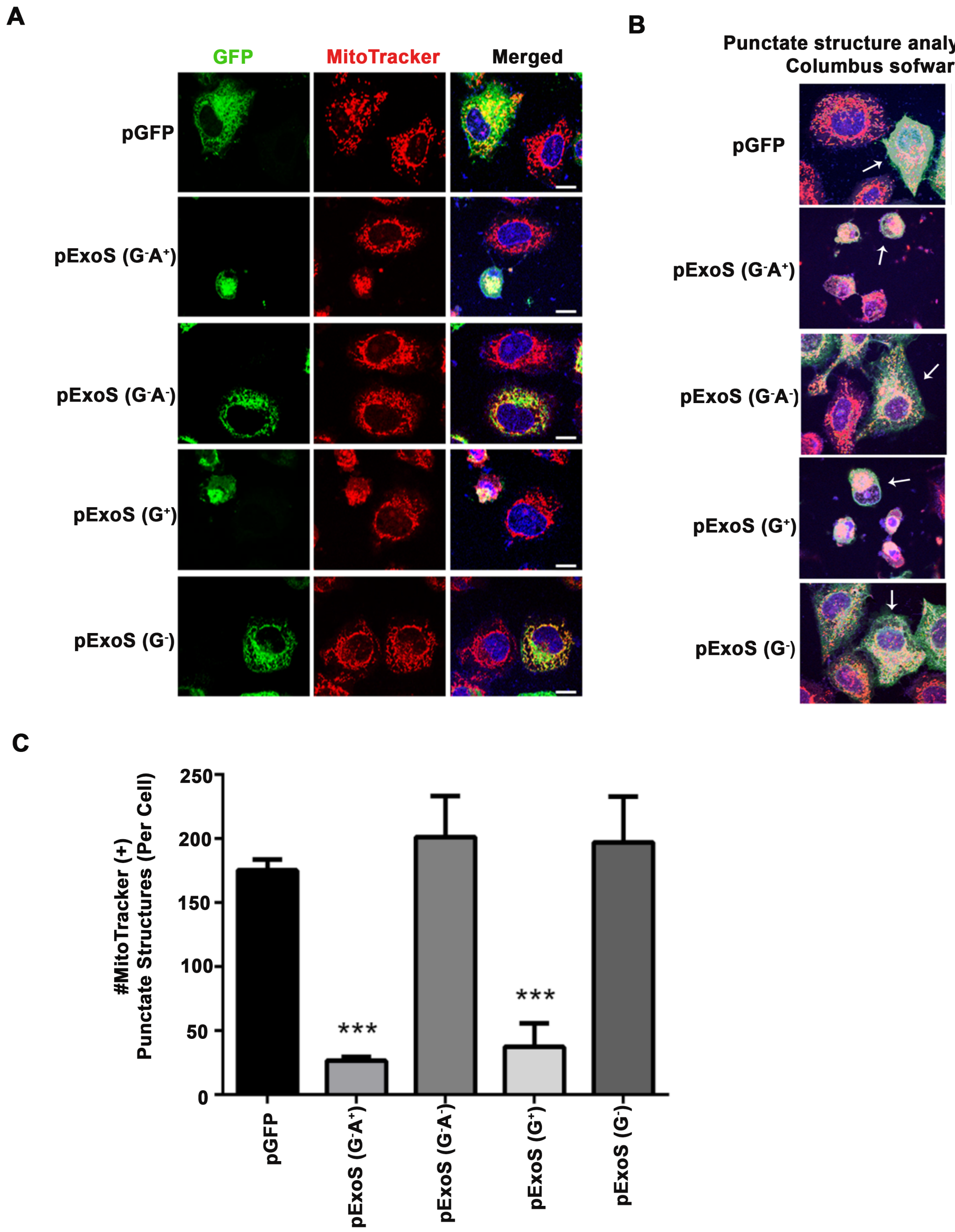

Figure S2. ExoS/GAP domain is sufficient to disrupt mitochondrial membrane as assessed by MitoTracker. HeLa cells were transfected with pIRES2-EGFP mammalian expression control vector (pGFP) or pIRES2 vectors containing full length or truncated GAP-expressing ExoS (pExoS (G^+A^-) or pExoS (G^+)), or their inactive GAP counterparts (pExoS (G^-A^-) or pExoS (G^-)), all directly fused to GFP at their C-termini. 20h after transfection, cells were fixed and stained for GFP (green), MitoTracker (red), and DAPI nuclear dye (blue), and the impact of ExoS/GAP on mitochondrial health was assessed by IF microscopy. **(A)** Representative images of MitoTracker stained transfected cells are shown. **(B-C)** Columbus software was used to outline and determine the number of intact mitochondria per cell (MitoTracker positive punctate structures represented by different colored circles). Representative Columbus images are shown in (**B**) and the tabulated data, as determined by Columbus software from 10 random fields/group, are shown as the Mean ± SD in (**C**) (*p < 0.01, one-way ANOVA). Scale bar represents 20μm.

SUPPLEMENTAL MOVIE LEGENDS

Movie S1. ExoS/GAP domain activity causes cytotoxicity in HeLa cells. HeLa cells were infected with **(A)** ΔUΔT/ExoS, **(B)** PA103 *pscJ* (*pscJ*), **(C)** pretreated with Z-LEHD-FMK for 1h, then infected with ΔUΔT/ExoS (G^+A^-), or **(D)** pretreated with caspase-3 inhibitor (Z-DEVD-FMK) for 1h, then infected with ΔUΔT/ExoS (G^+A^-) at a MOI of 10. Cytotoxicity was assessed by IF time-lapse videomicroscopy using propidium iodide (PI) uptake (red) as a marker for cell death, as described [13,21]. Video images were captured every 15 min for a period of 20h.

Movie S2. ExoS/GAP domain is sufficient to cause cytotoxicity in HeLa cells. HeLa cells were transfected with pIRES2 mammalian expression vector harboring: **(A)** full length ExoS with functional GAP and mutant ADPRT, directly fused to GFP at its C-terminus, pExoS (G^+A^-)-GFP; **(B)** Truncated ExoS with only functional GAP domain, directly fused to GFP at its C-terminus, pExoS (G^+)-GFP; **(C)** Full length ExoS with mutant GAP and mutant ADPRT domains, directly fused to GFP at its C-terminus, pExoS (G^-A^-)-GFP; **(D)** truncated ExoS with only mutant GAP domain, directly fused to GFP at its C-terminus, pExoS (G^-)-GFP; **(E)** pExoS (G^+A^-)-GFP in the presence of caspase-9 inhibitor (Z-LEHD-FMK); **(F)** pExoS (G^+)-GFP in the presence of caspase-9 inhibitor (Z-LEHD-FMK). Images were taken every 15 min and cytotoxicity was assessed by time-lapse videomicroscopy using propidium iodide (PI) uptake (red) as a marker for cell death as described [20,28].

Movie S3. Dynamics of ExoS/GAP-induced cytotoxicity in PA103 genetic background. HeLa cells were infected with: **(A)** ΔUΔT/ExoS, **(B)** ΔUΔT/ExoS (G^+A^-), **(C)** ΔUΔT/ExoS (G^-A^+), or **(D)** ΔUΔT/ExoS (G^-A^-) strains at a MOI of 10. Cytotoxicity was assessed by IF time-lapse videomicroscopy using propidium iodide (PI) uptake (red) as a marker for cell death, as described [13,21]. Video images were captured every 15 min for a period of 20h.

Movie S4. Dynamics of ExoS-induced cytotoxicity in PAK genetic background. HeLa cells were infected with: **(A)** ΔSΔT/ExoS, **(B)** ΔSΔT/ExoS (G^+A^-), **(C)** ΔSΔT/ExoS (G^-A^+), **(D)** ΔSΔT/ExoS (G^-A^-), or **(E)** PAK *pscJ*, (*pscJ*), at a MOI of 10. Cytotoxicity was assessed by IF time-lapse videomicroscopy using propidium iodide (PI) uptake (red) as a marker for cell death, as described [13,21]. Video images were captured every 15 min for a period of 20h.
